# Daily activity avoidance in the context of urinary and fecal incontinence among young adults with Spina Bifida: Preliminary results from an ecological momentary assessment study

**DOI:** 10.1371/journal.pone.0341057

**Published:** 2026-02-24

**Authors:** Devon J. Hensel, Audrey I. Young, Konrad M. Szymanski

**Affiliations:** 1 Division of Adolescent Medicine, Department of Pediatrics, Indiana University School of Medicine, Indianapolis, Indiana,; 2 Department of Biology, DePauw University, Greencastle, Indiana; 3 Division of Pediatric Urology, Riley Hospital for Children at Indiana University Health, Indianapolis, Indiana; Cranfield University, UNITED KINGDOM OF GREAT BRITAIN AND NORTHERN IRELAND

## Abstract

Although studies suggest that young adults with spina bifida (YASB) often avoid important daily activities (ADL) due to urinary (UI) and fecal (FI) incontinence, no research has prospectively examined how often ADL avoidance occurs, or the context in which it occurs. In this paper, we used ecological momentary assessment (EMA) over 30 days to describe: 1) the frequency of avoidance; 2) the activities YASB most commonly avoid; and 3) preliminary association of daily avoidance with affect, incontinence anxiety and health-related quality of life (HRQoL). As part of a larger 30-day prospective study designed to understand the incontinence in adults with SB (N = 89), participants completed an end-of-day EMA tracking daily ADL avoidance on days when they were worried about possible UI or FI and on days when they had actual UI or FI. Additional day-level measures were: affect and incontinence anxiety. HRQoL was reported in beginning- and end-of-study surveys. We drew a subsample of YASB participants (18–27 years; N = 23 of total 88 participants). ADL avoidance was most frequent with actual FI (20.5%) and least frequent with worry about FI (3.5%, p = 0.02) (UI worry: 8.3; actual UI: 8.9%, p = 0.546). The most common ADL avoided were eating, drinking and spending time with family/friends. Negative mood and incontinence anxiety were significantly higher on days with all types of avoidance. Higher baseline HRQoL was associated with fewer in-study ADLs avoided, which were in turn associated with higher end-of-study HRQoL. Our data suggest that YASB avoid key activities of daily living (e.g., eating, drinking, seeing family/friends) both when they worry about possible incontinence that may but does not occur, and when they experience actual incontinence. Tailored interventions addressing actual incontinence and worry about possible incontinence incorporating daily mood or incontinence anxiety may minimize ADL avoidance and its impact on long-term HRQoL.

## Introduction

Urinary (UI) and fecal (FI) incontinence are common secondary health conditions among young adults with spina bifida (YASB). Large scale studies report past month UI prevalence between 50% and 75% and past month FI prevalence between 41% and 55% [1]. Many YASB report both UI and FI in the past month [2]. Experiencing *actual* UI or FI and/or worrying about *possible* UI or FI can generally interfere with YASB’s participation in different activities of daily living (ADL) [3–5]. ADL are routine but essential tasks that individuals must be able to perform without assistance in order to be able to live independently [6]. People born with SB now have a lifespan approaching healthy populations, [7] meaning that YASB will live with UI and FI for several decades. Because of this increased lifespan, SB care guidelines now recommend that YASB learn to self-manage as many ADL as possible, as early as possible [8,9]. Maximizing ADL participation as YASB live amidst actual incontinence or worry about possible incontinence offers an important means for them to integrate themselves – even for YASB who are partially or completely dependent on caregivers – more fully into society at large [9]. In addition, greater ADL independence could facilitate the transition to adult care for some YASB [10] and is likely to bolster health related quality of life (HRQoL) for all YASB [11]. As with many other chronic illnesses, both increased independence and increased HRQOL are central therapeutic goals for YASB.

Providing effective support to YASB to reduce ADL avoidance requires accurately capturing what constitutes “clinically significant” avoidance in daily life. Clinical experience and prior research indicate that urinary and fecal incontinence vary not only between individuals, but also within the same individual across days [2,12–15]. Yet, fundamental questions about daily experiences needed to support clinical decision making – for example, *how often* does ADL avoidance occur in daily life, and *which activities* are most often avoided? Do these factors *differ between UI and FI*, whether an individual *experiences actual incontinence or only worries about incontinence that may but does not occur*, and do they *differ depending on the context of the day*? – remain unaddressed in the extant literature. Moreover, our past cross-sectional work suggests that objective aspects of UI and FI are independently associated with lower HRQoL [1,16,17], it remains unclear whether higher baseline HRQoL buffers against daily activity avoidance, or whether repeated avoidance in daily life contributes to subsequent reductions in HRQoL. Capturing these day-level experiences is therefore essential for truly personalized continence care, because interventions to address ADL avoidance must be adaptable not only to which activities are avoided and when avoidance occurs, they also must acknowledge when how these patterns change over time [12]. Notably, individualized approaches that focus on these shifts been identified as a research priority by people living with spina bifida themselves [18].

In the current paper, we use ecological momentary assessment (EMA) to gather these necessary data. Current clinical practice asks YASB to self-summarize incontinence experiences over long periods of time [2]. These static estimates can be prone to recall bias, and they often obscure potentially important between-person differences in how ADLA is experienced [19–22]. Interventions based on these data tend towards “one size fits all,” which may not meet all ASB needs. For example, a clinician who see that two YASB report the same number of ADL activities avoided may falsely assume the same therapies will be effective for them, even though the circumstances surrounding the avoidance may be very different for each YASB. The most effective patient-centered interventions are those tailored from the experiences of YASB themselves. EMA is a prospective – or forward looking – person-centered data collection approach that reduces this measurement bias through brief-but-repeated “in the moment” reports of YASB’s daily experiences (e.g., ADL avoidance due to worry about or actual UI or FI) and the context (e.g., mood or feelings about incontinence) in which they occur [23]. Over time, these repeated snapshots coalesce to provide a more accurate portrait of ADLA occurrence *in real life* [24]. By identifying when activity avoidance occurs, which activities are affected, and whether avoidance reflects actual incontinence or anticipatory worry, EMA-derived data could enable more individualized, participation-focused management than is possible with global symptom severity measures alone. Excellent reviews of EMA for overall health behavior can be found in Persky, et. al, 2022 [25] and Morris et. al, 2020 [26]. A review of the advantages of EMA use in the SB population is available in Hensel et. al, 2023 [2], Szymanski et. al, 2024 [12], or Hensel et. al, 2025 [27].

Accordingly, the primary objectives of our paper were to provide preliminary data on:

1. How often YASB avoid ADL when they either experience *actual* UI and/or FI or when they are worried about *possible* UI and/or FI (Objective 1a) and the most commonly avoided activities in these situations (Objective 1b);
2. Associations between daily ADL avoidance and daily affect or incontinence anxiety (Objective 2);
3. Association between beginning-of-study HRQoL and daily ADL avoidance, as well as between daily ADL avoidance and end-of-study HRQoL (Objective 3).

## Methods

### Study design and participants

Participants were a young adults 18−27 years (N = 23; 26.1% of all study-participants [N = 88]) in a larger 30-day study (R21DK121355) study examining the feasibility of using EMA to understand daily incontinence experiences among adults with SB. All participants completed end-of-day EMAs that assessed UI- and FI-specific characteristics, including any ADL avoidance, and if relevant, the specific activities avoided. Additional methodological details in Hensel et. al, 2023 [2] and Szymanski et. al, 2024 [12]. The larger project was approved by the Institutional Review Board of Indiana University (#1907916729), and achieved excellent retention (97.5%), EMA completion (95.8%), data accuracy (98.6%). Participants rated acceptability high (>90% enjoyed the study, found protocol easy, felt supported, would participate again). Completion was not associated with either demographic characteristics or any health history, including incontinence. While we observed no reactivity effects in data completion, there were small changes in UI reporting (−2.1%) and positive mood (+1.4%) over the 30 days [2,12]. Participant data are available in Table 1.

**Table 1:** Demographic and background characteristics of young adults with Spina Bifida.

Characteristic	Percent (N) or Mean (SD); Median
Age	22.9 (2.31); 23.0
Birth-assigned sex (female)	78.3
Gender identity (female)	78.3
Sexual orientation	
Heterosexual	82.6
Gay or lesbian	0
Bisexual	13
Asexual	4.3
Something else	0
Race/ethnicity	
White	91.3
Black	8.7
American Indian or Alaska Native	0.0
Asian	0.0
Other	0.0
Multiple races	0.0
Hispanic or Latino (yes)	4.4
Annual household income	
< $20,000	20.0
$20,000 to $39,000	15.0
$40,000 to $59,000	10.0
$60,000 to $79,000	5.0
$80,000 to $100,000	5.0
$100,000 or more	15.0
Unsure	30.0
Highest education level completed	
Less than high school	0.0
High school degree or equivalent	39.3
Some college but no degree	17.3
Bachelor’s degree	34.7
Graduate degree	8.0
Current work status	
Working full time	17.3
Working part time	26.1
Unemployed and looking for work	17.3
Unemployed and not looking for work	13.0
On disability	26.1
Currently attending school (full or part times: yes)	17.3
Current relationship status	
Single and not dating	56.5
Single and dating but not in a relationship	8.7
In a relationship, but not living together	13.0
In a relationship, living together (not married)	8.7
Married	13.0
Divorced	0.0
Widowed	0.0
Currently living independently (yes)	39.3
Ambulation	
I move only in my wheelchair	26.1
I walk only during therapy. I use a wheelchair the rest of the time	4.3
I walk inside and outside (with or without a walker, braces or crutches). I only	17.4
use a wheelchair for long trips.	
I walk inside and outside without any help	26.1
Have a ventriculoperitoneal shunt (VPS) (yes)	60.8
Any incontinence at enrollment (past four weeks: yes)	100.0
UI only	17.4
FI only	13.0
Both UI and FI	69.6
Catheterize bladder (yes)	87.1
Malone antegrade colonic enema (MACE) to empty bowels (yes)	18.8

### Measures

#### ADL Avoidance.

Participants reported on any *activity avoidance with actual incontinence* (no/yes; separate item for UI and FI) and any *activity avoidance when worried about possible incontinence* (no/yes; separate item for UI and FI). An affirmative answer was followed by a question asking which of four basic (chores/housework, eating a meal/snack, drinking a beverage, going to a medical appointment) or ten instrumental (exercise, taking transportation, going to school/work, having sex, in person time with family/friends, doing a hobby, attending religious service, texted/called someone, watched TV/movies, took a nap/rested) activities they avoided (all: no/yes).

#### Daily affect.

We included positive mood (“happy” and “friendly”) and negative mood (“angry” and “irritable”) (PANAS; both additive indices of the two, 5-point Likert items [not at all to extremely]).[28]

#### Daily incontinence anxiety.

We used a single 4-point Likert- type items that asked: “To what extent did you feel anxious about [incontinence type] today?” (not at all or very slightly, a little, moderately, extremely).

#### Health-related quality of life.

In the enrollment and exit surveys of the study, participants completed the Health and Relationships (a person’s perception of health quality and connections with others) domain of the *Quality of Life Assessment in Spina Bifida for Adults*.[1]

### Analyses

We used descriptive statistics to characterize the daily frequency of ADL avoidance when YASB experience actual UI or FI or when they are worried about UI or FI (Objective 1a), as well as to identify the types of basic and instrumental ADL most commonly avoided (Objective 1b). Owing to the smaller number of EMAs available for analysis, comparisons between incontinence types were performed using a Wilcoxon Rank sum tests, which is a nonparametric equivalent for related samples [29]. We used nonparametric Fisher’s Exact tests to examine median differences in positive mood, negative mood and incontinence anxiety on days when ADL were and were not avoided [29] and we used Spearman rank correlation coefficient to examine the preliminary associations between HRQoL and ADL avoidance (Objective 2) [30]. Finally, we used Spearman’s Rank Coefficient to assess the correlation between HRQoL at the beginning of the study and ADL avoidance, and between ADL avoidance and HRQoL at the end of the study (Objective 3) [31,32]. All data used a Bonferroni to adjust *p*-values for multiple comparisons.

## Results

### Overall patterns

#### Overall daily urinary and fecal incontinence frequency.

About six out of every ten days were associated with a report of any type of incontinence (57.5%; 370/643). The majority of these reports (63.7%: 236/370) were UI only. About one in ten (9.3%: 22/370) were FI only, and a third (30.2%: 112/370). Any UI was reported on 54.1% (348/643) of all study days. FI was noted on 20.8% (134/643) of all study days (*p* < .001).

**Objective 1a – Describe the frequency of daily ADL avoidance among YASB when they actually experienced UI or FI and ADL avoidance on non-incontinence days when they were worried about possible UI or FI**

#### Urinary incontinence.

YASB reported avoiding ADL with relatively the same frequency – about eight percent – on days when they were worried about possible UI (8.3%: 25/295) as they did on days when they actually experienced UI (8.9%: 31/348; *p* = 0.546). This suggests slightly less than one out of every ten days with a real or perceived concern about UI involved the avoidance of at least one activity. As shown in Table 2, the median number of ADL avoided per day for both sources were one, ranging between no activities and two activities avoided around worry about UI and ranging between no activities and three activities avoided when UI occurred.

**Table 2:** Daily reports of activity of daily living avoidance with actual incontinence or worry about possible incontinence among young adults with Spina Bifida.

	Worry Avoidance Type											
	Worry about Urinary Incontinence			Actual Urinary Incontinence			Worry about Fecal Incontinence			Actual Fecal Incontinence		
Total ALDs avoided per day (median)	1			1			1			1		
Total ADLs avoided per day (range)	0 - 2			0 - 3			0 - 2			0 - 4		
Basic ADL (%)												
Chores/housework	0.0			12.9			5.6			11.1		
Eating a meal	44.0			16.1			55.6			55.6		
Drinking a beverage	88.0			67.7			44.0			22.2		
Instrumental ADL (%)												
Exercised	44.0			19.3			11.1			18.5		
Attending a medical appointment	0.0			0.0			0.0			3.7		
Took bus, train, car or plane	12.0			3.2			0.0			3.7		
Went to school or work	0.0			3.2			5.6			7.4		
Had sex	0.0			19.6			11.1			14.8		
Spent time with family or friends	4.0			12.9			27.8			33.3		
Did a hobby	8.0			9.6			5.6			11.1		
Went to religious service	0.0			3.2			0.0			3.7		
Texted, called someone or used the internet	0.0			0.0			0.0			0.0		
Watched TV or movie	0.0			0.0			0.0			3.7		
Took a nap or rested	16.0			6.5			5.6			3.7		
				
Day-Level Characteristics	Median		Wilcoxon *p-*value	Median		Wilcoxon *p-*value	Median		Wilcoxon *p-*value	Median		Wilcoxon *p-*value
	No		Yes		No		Yes		No	Yes	No	Yes
Positive mood (range: 2–10)	6.0	5.0	0.119	6.0	6.0	0.234	6.0	5.0	0.003	6.0	6.0	0.051
Negative mood (range: 2–10)	3.0	4.0	0.101	3.0	4.0	0.001	3.0	3.0	0.054	3.0	4.0	0.001
Incontinence anxiety (range: 1–5)	1.0	3.0	<.001	2.0	3.0	<.001	1.0	3.0	<.001	2.0	4.0	<.001

ADL = activity of daily living; CI = Confidence Interval.

#### Fecal incontinence.

YASB noted ADL about five times less frequently on days when they were worried about FI (3.5%: 18/509) versus days when they actually experienced FI (20.2%: 27/134; *p* = 0.002). These latter data suggest that one out of every five days with FI also involved activity avoidance. As shown in Table 2, the median number of ADL avoided per day for actual FI and worry about FI sources were one, ranging between no activities and two activities avoided around worry about FI and ranging between no activities and four activities avoided when FI occurred.

#### Supplementary analyses.

We explored different comparisons *across* worry types. On 112 days when YASB reported both actual UI and FI, 12.5% (14/112) were associated with ADL avoidance for UI only and 7.1% (8/112) involved avoiding ADL around both UI and FI. No days (0.0%) were ADL avoidance for FI only (*p* = 0.0125 across the three groups). On 273 days when neither UI nor FI were reported, 1.8% (5/273) were concurrent with avoiding activities for worry about UI and 10.2% (25/273) for worry about FI only. No days had activity avoidance around worry for both UI and FI (*p* = 0.664 across the three groups).

Daily ADL avoidance was significantly *more frequent* when YASB reported *actual* FI than when they reported *actual* UI (*p* < 0.001). Conversely, daily ADL avoidance was significantly *more frequent* on days when YASB *worried* about UI as compared to when they *worried* about FI (*p* < 0.001).

**Objective 1b – Describe the most commonly ADL YASB avoid they either experienced UI or FI or when they were worried about possible UI or FI**

Table 2 displays the frequency of both basic and instrumental ADL avoidance, by actual incontinence and worry about incontinence. Of basic ADL, the most broadly endorsed activities avoided were eating and drinking. Of instrumental activities, the most commonly avoided activities were spending time with family or friends. Avoidance of eating a meal or snack was most frequent on days with actual UI (44.0%), with actual FI (55.6%) or worry about FI (55.6%). Drinking was most commonly avoided on days with worry about possible UI (88.0%) or with actual UI (67.7%), with worry about FI (44.0%) or with actual FI (22.2%). YASB reported avoiding exercises the most frequently when they were worried about UI (44.0%) (actual UI: 19.3% and actual FI: 18.5%). Finally, spending time with family and friends was most common on days with either worry about possible FI (27.8%) or with worry about possible FI (33.3%).

**Objective 2 – Understand preliminary median differences in positive mood, negative mood and incontinence anxiety on days with and without any ADL.**

As shown in Table 3, median UI anxiety was significantly *higher* on days when YASB reported ADL avoidance (vs. no avoidance) due to either worry about possible UI *or* experiencing actual UI (both *p* < .001). Median FI anxiety was also significantly higher on days when YASB reported ADL avoidance (vs. no avoidance) when they worried about possible FI *or* when they experienced actual FI (both *p* < .001). Negative mood was significantly *higher* on days when YASB avoided ADL both due to actual UI and actual FI (both *p* < .001) and positive mood was significantly *lower* on days when youth avoided ADL because they were worried about possible FI (*p* = .003).

**Table 3:** Daily affect, incontinence anxiety and activity of daily living avoidance among young adults with Spina Bifida.

	Avoided any ADL – worried about UI			Avoided any ADL – actual UI		
	Median		Fisher’s Exact *p*-value	Median		Fisher’s Exact *p*-value
	No	Yes		No	Yes	
Positive mood (range: 2–10)	6.00	5.50	0.119	6.00	6.00	0.234
Negative mood (range: 2–10)	3.00	4.00	0.101	3.00In	4.00	0.001
Incontinence anxiety (range: 1–5)	1.00	3.00	<.001	2.00	3.00	<.001
	Avoided any ADL – worried about FI			Avoided any ADL – actual FI		
	Median		Fisher’s Exact *p*-value	Median		Fisher’s Exact *p*-value
	No	Yes		No	Yes	
Positive mood (range: 2–10)	6.00	5.00	0.003	6.00	6.0	0.051
Negative mood (range: 2–10)	3.00	3.00	0.054	3.00	4.00	0.001
Incontinence anxiety (range: 1–5)	1.00	3.00	<.001	2.00	4.00	<.001

ADL = Activity of Daily Living; UI = urinary incontinence; FI = fecal incontinence.

##### Objective 3 – Association of ADL with Health Related Quality of Life

Higher HRQoL at the *start of the study* was significantly correlated with a lower number of days *during the study* on which YASB avoided ADL related to actual UI (Spearman’s Rank [SR] = −0.226; *p* < .001), actual FI (SR = −0.163; *p* = 0.004) or worry about FI (SR = −0.190; *p* < .001). Starting HRQoL was not correlated with avoidance due to worry about UI. Put another way, higher HRQoL before beginning the study seemed to buffer – or reduce – the frequency of activity avoidance during the 30 days.

A higher number of days *during the study* on which YASB avoided ADL due to actual UI (SR = −0.309; *p* < .001) or actual FI (SR = −0.165; *p* = 0.003) was significantly correlated with lower HRQoL at the *end of the study.* Similarly, YASB who reported a higher number of ADL avoidance days due to worry about UI (SR = −0.214; *p* < .001) or worry about FI (SR = −0.335; *p* < .001) *during the study* reported significantly *lower* HRQoL at the *end of the study*. Put another way, increased activity avoidance – for whatever motivation – over the 30 days reduced their HRQoL at the end of the month.

## Discussion

A central goal of caring for YASB is understanding any “clinically significant” impact incontinence may have on the function in their lives, including avoiding important daily activities. In the current paper, we used EMA to capture these avoidance events with greater precision to how YASB experience them in “real life.” Ours is the first study to contribute preliminary understanding of how often ADL avoidance occurs in the context of worry about incontinence or actual incontinence, what activities YASB most commonly avoid, and explore the impact of mood, incontinence anxiety and HRQoL on activity avoidance. While previous studies broadly suggest that UI and FI create barriers to ADL participation [3–5], our data indicate that this daily avoidance may be even more heterogenous that previously thought. While additional data will be needed to optimize EMA use in this population, below we highlight some preliminary examples of actionable insights that could enhance targeted clinical decision making rather than replacing routine assessment.

The first important idea is that both the overall occurrence of ADL avoidance and the specific activities YASB avoided varied depending on the both the incontinence source (e.g., UI vs. FI) and the avoidance source (e.g., worry about possible vs. actual incontinence). In other words, a dry day is not a day free of the negative effects of incontinence even though no incontinence occurs. While these statements may seem obvious, it is important to consider that most standardized clinical instruments screen for a single “usual” or “typical” actual incontinence outcome and do not capture worry about possible incontinence on days it does not occur [33,34]. Such an approach is unlikely to capture the fluctuation we observed over a 30-day period and ignore the effects of worry about potential incontinence. For example, if we consider that the median number of ADL avoided per day – one activity – is the same across incontinence source and avoidance source, we could falsely assume that avoidance consistently occurs at low levels. However, a closer look at the data would suggest important nuances, such as that YASB avoid vital basic ADL like eating and drinking frequently, or that YASB often forgo exercise with worry about UI, but not with actual UI, or that YASB avoid social time with family and friends about six times as often when they have actual FI as compared to when they have actual UI. These pieces of information are likely important inputs for more personalized continence care [12]. While the ideal EMA cadence and collection time to support clinical decision making remains to be established, this is an area worthy of further investigation. The opportunity to increase life participation – if even on a single activity – is important for all YASB, regardless of function level [10,11].

We observed that daily subjective factors were associated with activity participation. Specifically, median daily negative mood and daily incontinence anxiety were significantly higher when YASB reported that they avoided activities in every incontinence situation: when they were worried about possible UI, when they were worried about FI, when they reported UI and when they reported FI. While studies suggest that depression and anxiety are common in people with SB, [35] the role of affect in the improvement and exacerbation of incontinence symptoms remains unknown. The idea of targeting daily emotions alongside treating incontinence itself – perhaps in real time as part of just-in-time interventions – is an intriguing patient-centered approach yet unused in clinical settings. It may also be possible to include strengthening emotional management as part of self-management strategies [36]. Clinicians may consider screening for mood to open discussions about ADL avoidance [37].

Finally, our data suggested a preliminary association of cumulative ADL during the study with both beginning- *and* end-of-study reports of HRQoL. Specifically, *higher* HRQoL at the beginning of the study was significantly associated with *lower* ADL avoidance during the study, and *fewer* days of ADL during the study were associated with significantly *higher* HRQoL at the end of the study. On the one hand, these findings could suggest that building HRQoL serves as an important buffering effect to ongoing ADL avoidance in daily life. On the other hand, it could also be that lower activity avoidance reflects better condition management, increasing HRQoL. Disentangling the mechanism of these effects and their iterative clinical potential will be the focus of our future research, particularly in terms of how HRQoL could be leveraged to manage both short- and long-term well-being in those with SB.

### Limitations

There are methodological limitations associated with our study. First, while some measures (e.g., affect) have been used in prior EMA research, some of our other measures have only been validated in this population in retrospective research [2,12]. An important focus of these prior analyses has been evaluating the extent to which such a conversion was possible. While the preliminary look good (e.g., day-to-day variation, high completion), we will need to evaluate these measures in long EMA studies. Second, we captured only 30 days of data from each YASB, which is likely too short of a time to observe the “typical cycle” of ADL avoidance. This limited data volume also limited our ability to explore how other day-level factors (for example, objective incontinence markers like frequency or volume) may have been associated with ADL avoidance. While future work is planned to establish the optimal study length, a longer period – perhaps over six to twelve months – may better reflect “real life” YASB avoidance patterns. It will be important to know whether a higher volume of specific missed activities – such as school or work – are more adversely impactful on well-being over time than others. Additional daily observations would also permit a more extensive analysis of how contextual factors – like frequency or amount of incontinence, or past avoidance habits – impact daily avoidance. Future studies will focus on assessing both what an optimal data capture frame might be, and whether this frame should occur in a specific context. Second, while we did assess avoidance across nineteen different activities, there may be other ADL (e.g., basic hygiene or getting dressed) impacted by incontinence that we did not include. Relatedly, if an ADL were infrequent in a YASB’s life, a lack of avoidance endorsement could have indicated lack of general participation rather than avoidance due to incontinence. While no list of activities will likely represent every single YASB, future work should consider using YASB input as part of study planning to ensure a robust set of choices. Third, ongoing research should consider assessment of day-level factors we were unable to include – such as pain [38] or adherence to urinary or bowel management guidelines – that could have impacted activity participation. This and other ecological influences are planned as part of future studies in this area.

There are also sample limitations associated with the current data. Participants were primarily cognitively intact and exhibited a relatively high level of independence (e.g., more education, more stably employed, community ambulation), perhaps reflecting the feasibility requirements of intensive daily self-report methodologies. Individuals with greater cognitive impairment or higher caregiver dependence – particularly those with executive functioning limitations – may experience distinct patterns of activity avoidance that are not captured here. It is important to note that the larger study was not designed to assess population-level prevalence, but rather to establish proof-of-concept that daily activity avoidance related to incontinence can be prospectively measured and meaningfully linked to affect and quality of life. In this regard, we do not expect that our sample will reflect general clinical population characteristics. Future work should focus on adapting EMA protocols (e.g., caregiver-assisted reporting, simplified interfaces, reduced burden designs) to extend applicability across the broader spectrum of spina bifida. Second, the larger feasibility study by design excluded individuals under 18 years. Inclusion of teenage participants are planned as part of future work, particularly given that the developmental roots of adult incontinence management – of which ADL avoidance is a part – are likely linked to experiences in adolescence. Third, all participants had a recent history of incontinence. We did not capture incontinence location (e.g., per stoma or urethra/anus), which could have impacted YASB activity avoidance.

## Conclusion

This study is the first to capture a snapshot of what activity avoidance around incontinence – due to either worry about possible incontinence or actual incontinence – among YASB. Our data suggest that avoidance experiences are likely more varied than originally thought, and perhaps more importantly, have associations with both daily affect and incontinence anxiety as well as longer term HRQoL. Longer studies will be certainly needed to both capture a wider section of YASB life, and to establish the ideal data volume to enhance clinical decision making, Nevertheless, we argue that the user-centered and minimally invasive structure of EMA is well suited for gathering data from all people with SB about how they navigate daily life with incontinence. In addition to symptom reporting, EMA could do double duty as a platform to encourage patient-clinician communication and to facilitate the delivery of ongoing, personalized therapies [39].

## Abbreviations

EMA: Ecological momentary assessment
SB: Spina Bifida
UI: urinary incontinence
FI: fecal incontinence
ADL: Activity of Daily Living
ADLA: Activity of Daily Living Avoidance
